# The risk of death after hospitalisation following intentional self‐poisoning: a retrospective observational study (PAVLOVA‐2)

**DOI:** 10.5694/mja2.70068

**Published:** 2025-10-08

**Authors:** Firouzeh Noghrehchi, Nicholas A Buckley, Rose Cairns

**Affiliations:** ^1^ The University of Sydney Sydney NSW; ^2^ NSW Poisons Information Centre Children’s Hospital at Westmead Sydney NSW

**Keywords:** Overdose, Self‐injurious behavior, Epidemiology, Suicide, Toxicology, Mortality

## Abstract

**Objectives:**

To estimate the risk of death after hospitalisation with non‐fatal intentional self‐poisoning in New South Wales, and to estimate the associated number of years of life lost.

**Study design:**

Retrospective observational study; analysis of Poisoning And enVenomation Linkage to evaluate Outcomes and clinical Variation in Australia (PAVLOVA) study data.

**Setting, participants:**

All index admissions to New South Wales public and private hospitals of people after non‐fatal intentional self‐poisoning (ie, were discharged from the index admission alive), 1 January 2011 – 30 September 2020.

**Main outcome measures:**

Standardised mortality ratio (compared with general population mortality rate; SMR), overall, and by cause of death (data available only for 2011–2018); years of life lost (YLL) overall, and by cause of death (2011–2018), age group, and sex.

**Results:**

Index admissions of people with non‐fatal intentional self‐poisoning were identified for 48 951 people; their median age was 32.8 years (interquartile range [IQR], 20.8–47.5 years), 30 274 were girls or women (61.8%), and 3449 died during follow‐up (median, 4.9 years; IQR, 2.7–7.3 years). The all‐cause SMR was 3.1 (95% confidence interval [CI], 3.0–3.2); by cause of death, the SMR was highest for external cause deaths (16.8; 95% CI, 15.9–17.8), including accidental poisoning (30.3; 95% CI, 27.4–33.2) and suicide deaths (25.1; 95% CI, 23.2–27.1). Among natural causes of death, the SMR was highest for infectious and parasitic diseases (5.4; 95% CI, 3.9–6.8), digestive diseases (4.2; 95% CI, 3.4–5.0), and respiratory diseases (3.0; 95% CI, 2.5–3.4). The estimated overall premature mortality burden was 110 301.4 YLL; the median value per death was similar for women (31.1 YLL; IQR, 15.0–43.0 YLL) and men (33.2 YLL; IQR, 19.7–44.9 YLL). During 2011–2018, the total mortality burden was 79 821.6 YLL; by cause of death, the major contributors were deaths from suicide (26 945.2 YLL; 33.8%), accidental poisoning (17 436.1 YLL; 21.8%), other injuries (6026.8 YLL; 7.5%), and natural causes (29 413.5 years; 36.8%).

**Conclusions:**

The risk of death is markedly higher after hospitalisation with intentional self‐poisoning than for the general population, but suicide deaths only cause about one‐third of the mortality burden in terms of lost years of life; deaths from accidental poisoning and natural causes are also major contributors. Referrals to specialist psychiatric and physical health care and brief interventions for treating psychiatric and substance use conditions are appropriate after hospitalisation with intentional self‐poisoning.

**The known**: Intentional self‐poisonings are increasingly frequent in Australia, but longer term outcomes after discharge from hospital have not been examined.

**The new**: The risk of death during follow‐up (median, 4.9 years) for people hospitalised after poisoning themselves in New South Wales during 2011–2020 was three times as high as for the general population; it was particularly high for death by suicide (25 times as high) or accidental poisoning (30 times). However, only one‐third of the years of life lost were associated with suicide deaths.

**The implications**: Standard aftercare for people who have poisoned themselves should include both mental health assessments, including of substance use problems, as well as reviews of physical health conditions.

Intentional self‐harm is associated with increased risk of premature death.^1^ Most studies have focused on the risk of suicide after intentional self‐harm, which is particularly high soon after non‐fatal events.^2^, ^3^, ^4^, ^5^ Less well characterised is the risk of death from natural causes and accidental injuries for people who intentionally harm themselves.^1^, ^6^, ^7^ A 2012 United Kingdom study found that deaths from accidental poisonings were 25 times as frequent as in the general population after non‐fatal intentional self‐harm events, and deaths from natural causes 2.0–7.5 times as frequent.^1^

Rates of intentional self‐harm by young girls have increased in recent years in many countries.^8^, ^9^, ^10^, ^11^, ^12^, ^13^, ^14^ Intentional self‐poisoning is the most frequent cause of self‐harm leading to hospitalisation.^9^ The peak age for intentional self‐poisoning is moving to younger age groups,^9^ which may lead to increased excess mortality from suicide, injury, and natural causes.

Given recent changes in the epidemiology of intentional self‐poisoning, current information on long term outcomes is important, including assessments of the associated disease burden and premature mortality. We therefore investigated the risk of premature death after hospitalisation with non‐fatal intentional self‐poisoning in a large, multicentre New South Wales data linkage study. Specifically, we assessed all‐cause and cause‐specific mortality after intentional self‐poisoning, by age group and sex; estimated the impact of time on excess mortality; and estimated the number of years of life lost, by broad cause of death.

## Methods

For our retrospective observational study we analysed data from the Poisoning And enVenomation Linkage to examine Outcomes and clinical Variation in Australia (PAVLOVA) study, a large longitudinal data linkage study undertaken in New South Wales during 1 January 2011 – 30 September 2020.^15^ We report our study according to the Reporting of studies Conducted using Observational Routinely‐collected health Data (RECORD) checklist.^16^

For our analysis we used a subset of the PAVLOVA dataset, linked hospital admissions (Admitted Patient Data Collection, APDC) and deaths data. The APDC comprises data for hospital admissions to New South Wales public and private hospitals, including admissions to emergency department short stay units; each APDC admission record can include up to 51 diagnosis codes.^17^ We included data for people for whom a non‐fatal intentional self‐poisoning event was recorded in the APDC, defined by the International Classification of Diseases, tenth revision, Australian modification (ICD‐10‐AM) external cause codes X60–X69 in any diagnosis field. These records were linked to deaths information, including fact of death in the Registry of Births, Deaths and Marriages dataset and cause of death in the Cause of Death Unit Record File (COD‐URF).^18^ As at the time of linkage official cause of death data were available only to 31 December 2018, only fact of death information was available for later data points; consequently, we estimated all‐cause mortality for the entire cohort, but cause‐specific mortality for the period 1 January 2011 – 31 December 2018. The COD‐URF has fields for underlying and multiple contributing causes of death. The underlying cause of death is that which initiated the train of events leading to death; contributing causes are other medical conditions, diseases, and injuries associated with the death. We used the underlying cause of death for the cause‐specific death analysis, consistent with national statistical practice.

Sex as recorded in the APDC was used for our analyses; it is defined in the APDC data dictionary as “the biological sex of the patient”,^6^ but the field may include sex or gender information.

Probabilistic data linkage was performed by the New South Wales Health Centre for Health Records Linkage (CHeReL) using ChoiceMaker software (https://www.choicemaker.com).

### Statistical analysis

We defined the index event as being the first intentional self‐poisoning hospital admission during 1 January 2011 – 30 September 2020. Study exit date was defined as the end of follow‐up or death, whichever came first. We summarise variable data as numbers and proportions for categorical variables or medians and interquartile ranges (IQR) for non‐normally distributed continuous variables.

We calculated the Charlson Comorbidity Index score for each person using diagnosis codes in the APDC,^19^, ^20^ including admissions from across the entire dataset (ie, not restricted to the index admission). We used diagnosis codes (ICD‐10‐AM F01–F99) to identify additional psychiatric and substance use disorders. For our descriptive analysis of deaths by cause, we used categories based on ICD‐10‐AM codes (Supporting Information, table 1).

We calculated standardised mortality ratios (SMR) using indirect standardisation, adjusted for sex, age group (five‐year bands), and calendar year (one‐year bands). The SMR is a measure of relative excess mortality; SMR = 1 indicates that the mortality rate for the study population is the same as that for the general population. General population mortality rates were calculated using national statistics in the Australian Institute of Health and Welfare General Record of Incidence of Mortality (GRIM) books,^21^ which provide mortality data by sex, age, calendar year, and cause of death. Study population mortality rates were estimated with the Kaplan–Meier method, stratified by sex, age group, and calendar year; key assumptions were assessed by visual inspection of Kaplan–Meier curves. The number of person‐years at risk were based on the time from the index event to the date of study exit; this period was used to adjust for individual time at risk, as the duration of follow‐up differed between individuals. We calculated all‐cause SMR and cause‐specific SMRs based on ICD‐10‐AM chapters (first character level); for each cause‐specific SMR, we censored deaths from competing causes.^22^ External cause deaths (chapter 20) were subdivided into three‐character level blocks (Supporting Information, table 1). We calculated SMRs for each month of the first year after the index event and for each year thereafter. We calculated exact 95% confidence intervals (CI) for SMRs by assuming a Poisson distribution for the number of deaths and fixed person‐years at risk.

We calculated years of life lost (YLL) as the difference between age at the time of death and general population life expectancy, matched for age, sex, and calendar year, using Australian Bureau of Statistics life tables.^23^ The mean YLL per death was calculated for each group.

All analyses were undertaken in R 4.3.3, using the *popEpi*, *survival*, and *survminer* packages for SMR and Kaplan–Meier calculations.

### Ethics approval

This study was approved and the requirement for individual consent for access to personal data waived by the New South Wales Population and Health Services Human Research Ethics Committee (2019‐ETH11677).

## Results

The PAVLOVA cohort comprised 49 259 people admitted to hospital with intentional self‐poisoning. After excluding 304 people for whom the index event was fatal, one whose sex was recorded as “other”, and three with death dates preceding the index admission (data entry errors or false linkages), 48 951 people were included in our analysis. Their median age was 32.8 years (IQR, 20.8–47.5 years); 30 274 (61.8%) were girls or women (median age, 29.9 years; IQR, 18.9–46.2 years) and 18 677 (38.2%) were boys or men (median age, 36.6 years; IQR, 24.9–48.9 years). The median follow‐up time was 4.9 years (IQR, 2.7–7.3 years). Admission dates for 41 406 people were earlier than 31 December 2018 (cut‐off date for cause of death data); the median follow‐up time for this group was 4.0 years (IQR, 2.0–5.9 years).

The median age of people who had died by 30 September 2020 (52.9 [IQR, 39.6–69.8] years) was higher than for those still living (31.5 [IQR, 20.3–45.9] years); larger proportions had been hospitalised after poisonings involving pesticides, gases, or chemicals (6.2% *v* 5.3%) or required intensive care unit admission (20.2% *v* 12.5%), and their median index hospital stay was longer (4 [IQR, 1–11] *v* 2 [IQR, 1–4] days). The proportions of people with mental disorders caused by physiological conditions (26.7% *v* 6.5%) or psychoactive substance use (58.3% *v* 43.5%), schizophrenia (15.6% *v* 10.1%), or mood disorders (63.1% *v* 54.0%) were larger for people who had died than for those still living at study exit (Box 1).

Box 1 **Characteristics of 48 951 people at the time of index intentional self‐poisoning event, PAVLOVA cohort, New South Wales, 2011–2020, by outcome at the end of the follow‐up period**

**Table:** 

Characteristic	Still living	Died	Total
All people	45 502 [93.0%]	3449 [7.0%]	48 951
Sex			
Female	28 717 (63.1%)	1557 (45.1%)	30 274 (61.8%)
Male	16 785 (36.9%)	1892 (54.9%)	18 677 (38.2%)
Age (years) median (IQR)	31.5 (20.3–45.9)	52.9 (39.6–69.8)	32.8 (20.8–47.5)
Age group (years)			
Under 15	2005 (4.4%)	15 (0.4%)	2020 (4.1%)
15–24	14 905 (32.8%)	223 (6.5%)	15 128 (30.9%)
25–34	8635 (19.0%)	387 (11.2%)	9022 (18.4%)
35–44	7819 (17.2%)	594 (17.2%)	8413 (17.2%)
45–54	6588 (14.5%)	655 (19%)	7243 (14.8%)
55–64	3302 (7.3%)	514 (14.9%)	3816 (7.8%)
65–74	1399 (3.1%)	400 (11.6%)	1799 (3.7%)
75–84	630 (1.4%)	367 (10.6%)	997 (2.0%)
85 or older	219 (0.5%)	294 (8.5%)	513 (1.0%)
Means of index self‐poisoning (ICD‐10‐AM codes)*			
Drugs (X60–X64)	43 659 (95.9%)	3271 (94.8%)	46 930 (95.9%)
Alcohol (X65)	8657 (19.0%)	597 (17.3%)	9254 (18.9%)
Solvents, gases, vapours (X66, X67)	882 (1.9%)	91 (2.6%)	973 (2.0%)
Pesticides (X68)	327 (0.7%)	33 (1.0%)	360 (0.7%)
Other and unspecified (X69)	1214 (2.7%)	91 (2.6%)	1305 (2.7%)
Admission to intensive care unit	5676 (12.5%)	695 (20.2%)	6371 (13.0%)
Duration of hospital stay (days), median (IQR)	2 (1–4)	4 (1–11)	2 (1–5)
Charlson Comorbidity Index score, median (IQR)	0 (0–1)	3 (0–7)	0 (0–1)
Psychiatric conditions (ICD‐10‐AM codes)			
Mental disorders due to known physiological conditions (F01–F09)	2957 (6.5%)	923 (26.7%)	3880 (7.9%)
Mental and behavioural disorders due to psychoactive substance use (F10–F19)	19 794 (43.5%)	2015 (58.3%)	21 809 (44.5%)
Schizophrenia, schizotypal, delusional, and other non‐mood psychotic disorders (F20–F29)	4602 (10.1%)	541 (15.6%)	5143 (10.5%)
Mood (affective) disorders (F30–F39)	24 561 (54.0%)	2181 (63.1%)	26 742 (54.6%)
Anxiety, dissociative, stress‐related, somatoform and other nonpsychotic mental disorders (F40–F48)	24 816 (54.5%)	1855 (53.7%)	26 671 (54.5%)
Behavioural syndromes associated with physiological disturbances and physical factors (F50–F59)	2283 (5.0%)	120 (3.5%)	2403 (4.9%)
Disorders of adult personality and behaviour (F60–F69)	12 029 (26.4%)	809 (23.4%)	12 838 (26.2%)
Intellectual disabilities (F70–F79)	712 (1.6%)	50 (1.4%)	762 (1.6%)
Pervasive and specific developmental disorders (F80–F89)	816 (1.8%)	34 (1.0%)	850 (1.7%)
Behavioural and emotional disorders with onset usually occurring in childhood and adolescence (F90–F98)	2539 (5.6%)	139 (4%)	2678 (5.5%)
Unspecified mental disorder (F99)	457 (1.0%)	36 (1.0%)	493 (1.0%)
Marital status			
Married/de facto	11 978 (26.3%)	1068 (31%)	13 046 (26.7%)
Never married	25 959 (57.1%)	1216 (35.3%)	27 175 (55.5%)
Widowed	1477 (3.2%)	462 (13.4%)	1939 (4.0%)
Divorced	2731 (6.0%)	415 (12.0%)	3146 (6.4%)
Separated	2539 (5.6%)	240 (7.0%)	2779 (5.7%)
Unknown/blank/declined to respond	818 (1.8%)	48 (1.4%)	866 (1.8%)
Discharge			
Discharged by hospital	40 281 (88.5%)	2789 (80.9%)	43 070 (88.0%)
Discharged at own risk	1709 (3.8%)	202 (5.9%)	1911 (3.9%)
Transferred to nursing home	75 (0.2%)	94 (2.7%)	169 (0.3%)
Transferred to psychiatric hospital	1480 (3.3%)	129 (3.7%)	1609 (3.3%)
Transferred to hospice, palliative care or other accommodation	196 (0.4%)	35 (1.0%)	231 (0.5%)
Type change separation	585 (1.3%)	71 (2.1%)	656 (1.3%)
Discharge on leave	257 (0.6%)	27 (0.8%)	284 (0.6%)
Unknown/blank	919 (2.0%)	102 (2.9%)	1021 (2.1%)

ICD‐10‐AM = International Classification of Diseases, tenth revision, Australian modification; IQR = interquartile range; PAVLOVA = Poisoning And enVenomation Linkage to examine Outcomes and clinical Variation in Australia.

* Multiple means can be listed for a single event.

### Deaths during follow‐up

A total of 3449 of 48 951 people died during follow‐up (7.0%); among the 2468 deaths by 31 December 2018, 1265 were natural cause deaths and 1203 external cause deaths (644 suicide deaths, 559 other external cause deaths) (Box 2). Median age at death was 55.8 years (IQR, 42.4–72.4 years); 1101 deaths were of girls or women (median age, 57.5 [IQR, 43.8–76.0] years), 1367 of boys or men (median age, 54.5 [IQR, 41.2–70.0] years). The proportions of suicide (27.3% *v* 24.6%) and other external cause deaths (24.5% *v* 20.3%) were larger for male than female former patients. The median age at death was lower for suicide deaths (41.8 years; IQR, 29.9–53.3 years) than for natural cause deaths (65.0 years; IQR, 52.0–79.5 years).

The 644 suicide deaths included 415 involving method switching (ie, suicide by means other than poisoning); the most frequent suicide method recorded was intentional self‐harm by hanging, strangulation, and suffocation (X70; 286 deaths) (Supporting Information, table 2). Method switching was more frequent among male (252 of 373, 65.6%) than female former patients (163 of 270, 60.4%) (Supporting Information, table 3).

Box 2 **Deaths following index hospitalisations with non‐fatal intentional self‐poisoning, PAVLOVA cohort, New South Wales, 1 January 2011 – 31 December 2018, by age group, cause of death (ICD‐10‐AM codes), and sex**

**Table:** 

	Suicide (X60–X84)		Other external causes (V01–X59, X85–Y98)		Natural causes (A00–R99)		All deaths	
Age group (years)	Male	Female	Male	Female	Male	Female	Male	Female
All ages	373	271	335	224	659	606	1367	1101
Under 15*	< 5	< 5	< 5	< 5	< 5	< 5	< 5	†
15–24	61	37	30	15	< 5	7	†	59
25–34	69	53	80	41	28	18	177	112
35–44	85	57	108	72	63	62	256	191
45–54	76	66	67	58	110	105	253	229
55–64	41	26	31	22	132	102	204	150
65–74	23	17	9	9	129	97	161	123
75–84	10	11	5	< 5	115	108	130	†
85 or older	8	< 5	< 5	< 5	77	106	89	108

ICD‐10‐AM = International Classification of Diseases, tenth revision, Australian modification; PAVLOVA = Poisoning And enVenomation Linkage to examine Outcomes and clinical Variation in Australia.

* Total for people less than 15 years of age: nine deaths.

† Number is five or more, but cell count suppressed to prevent calculation of counts of less than five from being calculated.

### Standardised mortality ratios

The all‐cause SMR was 3.1 (95% CI, 3.0–3.2); it was higher for male (3.5; 95% CI, 3.3–3.6) than female former patients (2.7; 95% CI, 2.5–2.8). The SMR was highest for external cause deaths (16.8; 95% CI, 15.9–17.8), including accidental poisoning (30.3; 95% CI, 27.4–33.2) and suicide deaths (25.1; 95% CI 23.2–27.1) (Box 3). All‐cause SMR values declined with age; within age groups they were higher for male than female former patients (Box 4).

Among natural causes of death, the SMRs were highest for infectious and parasitic diseases (5.4; 95% CI, 3.9–6.8), digestive diseases (4.2; 95% CI, 3.4–5.0), and respiratory diseases (3.0; 95% CI, 2.5–3.4) (Box 3). The most frequent natural causes of death were other chronic obstructive pulmonary disease (J44; 99 deaths), chronic ischaemic heart disease (I25; 93 deaths), malignant neoplasm of bronchus and lung (C34; 74 deaths), acute myocardial infarction (I21; 52 deaths), and alcoholic liver disease (K70; 46 deaths).

Box 3 **Mortality during follow‐up after index hospital admission following non‐fatal intentional self‐poisoning, PAVLOVA cohort, New South Wales, 1 January 2011 – 31 December 2018: standardised mortality ratios (SMR), by cause of death (ICD‐10‐AM code group) and sex**

**Table:** 

	Male former patients			Female former patients			All people		
	Deaths			Deaths			Deaths		
Cause of death (ICD‐10‐AM)	Actual	Expected	SMR (95% CI)	Actual	Expected	SMR (95% CI)	Actual	Expected	SMR (95% CI)
**All causes (fact of death data, 2011–2020)**	1892	544.9	3.5 (3.3–3.6)	1557	581.7	2.7 (2.5–2.8)	3449	1126.6	3.1 (3.0–3.2)
**All causes (cause of death data, (2011–2018)**	1367	328.8	4.2 (3.9–4.4)	1101	351.6	3.1 (2.9–3.3)	2468	680.3	3.6 (3.5–3.8)
**Natural causes**	659	298	2.2 (2.0–2.4)	606	332	1.8 (1.7–1.9)	1265	629	2.0 (1.9–2.1)
Certain infectious and parasitic diseases (A00–B99)	30	4.9	6.1 (3.9–8.3)	25	5.4	4.7 (2.8–6.5)	55	10.3	5.4 (3.9–6.8)
Neoplasms C00–D48	166	105.5	1.6 (1.3–1.8)	157	108.8	1.4 (1.2–1.7)	323	214.3	1.5 (1.3–1.7)
Diseases of the blood and blood‐forming organs and certain disorders involving the immune mechanism (D50–D89)	< 5	—	—	< 5	—	—	6	2.2	2.7 (0.5–4.9)
Endocrine‐nutritional and metabolic disorders (E00–E90)	39	13.7	2.8 (1.9–3.7)	35	14.4	2.4 (1.6–3.2)	74	28.1	2.6 (2–3.2)
Mental and behavioural disorders (F00–F99)	22	12.8	1.7 (1.0–2.4)	27	24.2	1.1 (0.7–1.5)	49	37.0	1.3 (1–1.7)
Diseases of the nervous system (G99)	32	15.7	2 (1.3–2.7)	31	20.9	1.5 (1.0–2.0)	63	36.6	1.7 (1.3–2.1)
Diseases of the circulatory system (I00–I99)	186	82.9	2.2 (1.9–2.6)	152	89.4	1.7 (1.4–2.0)	338	172.3	2.0 (1.8–2.2)
Diseases of the respiratory system (J00–J99)	82	26.8	3.1 (2.4–3.7)	87	30.1	2.9 (2.3–3.5)	169	57.0	3.0 (2.5–3.4)
Diseases of the digestive system (K00–K93)	65	13.4	4.9 (3.7–6)	49	13.9	3.5 (2.5–4.5)	114	27.2	4.2 (3.4–5.0)
Diseases of the musculoskeletal systems and connective tissue (M00–M99)	5	1.9	2.7 (0.3–5.0)	6	3.8	1.6 (0.3–2.8)	11	5.7	1.9 (0.8–3.1)
Diseases of the genitourinary system (N00–N99)	6	5.7	1.1 (0.2–1.9)	10	7.7	1.3 (0.5–2.1)	16	13.4	1.2 (0.6–1.8)
Symptoms, signs, and abnormal clinical and laboratory findings not elsewhere classified (R00–R99)	21	3.0	7.1 (4.1–10.1)	20	3.9	5.1 (2.9–7.3)	41	6.9	5.9 (4.1–7.8)
**External causes of morbidity and mortality**	708	43.5	16.3 (15.1–17.5)	495	28.0	17.7 (16.1–19.3)	1203	71.4	16.8 (15.9–17.8)
Suicide (X60–X84)	373	17.1	21.9 (19.6–24.1)	271	8.5	31.7 (27.9–35.5)	644	25.6	25.1 (23.2–27.1)
Accidental poisoning (X40–X49)	233	8.4	27.8 (24.2–31.3)	174	5.0	34.5 (29.4–39.7)	407	13.4	30.3 (27.4–33.2)
Assault (X85–Y09)	*	—	—	< 5	—	—	13	2.0	6.6 (3.0–10.1)
Accidental drowning (W65–W74)	7	0.8	9 (2.3–15.7)	0	0.3	0	7	1.1	6.3 (1.6–11.0)
Land transport accidents (V01–V89)	31	6.2	5.0 (3.2–6.7)	9	3.2	2.8 (1.0–4.6)	40	9.5	4.2 (2.9–5.5)

ICD‐10‐AM = International Classification of Diseases, tenth revision, Australian modification; IQR = interquartile range; PAVLOVA = Poisoning And enVenomation Linkage to examine Outcomes and clinical Variation in Australia.

* Number is five or more, but cell count suppressed to prevent calculation of counts of less than five from being calculated.

Box 4 Mortality during follow‐up after index hospital admission following non‐fatal intentional self‐poisoning, PAVLOVA cohort, New South Wales, 1 January 2011 – 30 September 2020: standardised mortality ratios (SMR), by age group and sex*

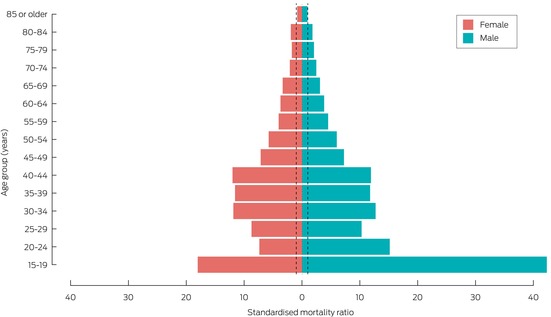

PAVLOVA = Poisoning And enVenomation Linkage to examine Outcomes and clinical Variation in Australia.

* Deaths of people under 15 years of age not included because of small numbers. Dotted lines mark SMR = 1 (comparative population level).

The all‐cause SMR was highest during the year after the index admission (5.5; 95% CI, 1.2–10.7) (Box 5), particularly during the first (SMR, 8.4; 95% CI, 2.7–14.0) and second months (SMR, 7.6; 95% CI, 2.2–13.0) after the index admission (Supporting Information, figure 1). Most of the increased mortality during the first year was attributable to external cause deaths (Box 5), particularly during the first month after the index admission (SMR, 62.0; 95% CI, 46.6–77.4) (Supporting Information, figure 1). Most external cause deaths during the first year were suicide (SMR, 60.4; 95% CI, 45.2–75.6) and accidental poisoning deaths (SMR, 49.8; 95% CI, 36.0–63.6) (Box 5).

Box 5 Mortality during follow‐up after index hospital admission following non‐fatal intentional self‐poisoning, PAVLOVA cohort, New South Wales, 1 January 2011 – 31 December 2018: standardised mortality ratios (SMR), by cause of death and time since index admission (years)*

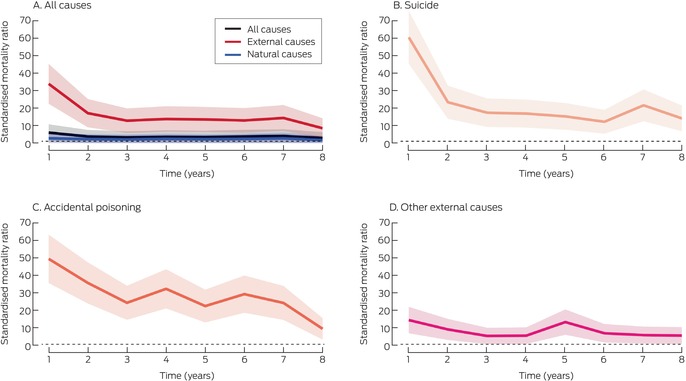

PAVLOVA = Poisoning And enVenomation Linkage to examine Outcomes and clinical Variation in Australia.

* Shaded areas: 95% confidence intervals; dotted lines mark SMR = 1 (comparative population level). The corresponding graphs by month during the first year after the index hospitalisation are included in the Supporting Information, figure 1.

### Years of life lost

We estimated that the overall premature mortality burden was 110 301.4 YLL. The median value per death was 31.8 YLL (IQR, 17.2–44.0 YLL); it was similar for female (31.1 YLL; IQR, 15.0–43.0 YLL) and male former patients (33.2 YLL; IQR, 19.7–44.9 YLL). The total premature mortality burden for deaths to 31 December 2018 was 79 821.6 YLL; the main contributors were external causes (50 408.1 YLL; 63.2%), including suicide (26 945.2 YLL; 33.8%), accidental poisoning (17 436.1 YLL; 21.8%), other injuries (6026.8 YLL; 7.5%), and natural causes (29 413.5 years; 36.8%). The mortality burden for suicide (11 508.4 *v* 15 436.8 YLL) and accidental poisoning (4328.7 v 13 107.4 YLL) were each lower for female than male former patients (Supporting Information, figure 2). The main natural cause contributors were neoplasms (7222.2 YLL; 9.0%), and circulatory system diseases (6582.8 YLL; 8.2%) (Supporting Information, figure 3). The total premature mortality burden attributable to external causes declined with age in both sexes (Supporting Information, figures 4 and 5).

## Discussion

We found that mortality risk is greater for people after hospitalisation following intentional self‐poisoning than for the general population, particularly the risks of death from accidental poisoning and suicide; the risk of death from natural causes was also higher. About 7% of people hospitalised following deliberate self‐poisoning died during follow‐up (median, 4.9 years), and the risk was greatest during the year following the index event.

The cohort in our study was larger than in other investigations that have estimated SMRs during longer term follow‐up after intentional self‐poisoning (more than one year).^1^, ^5^, ^6^, ^7^, ^13^, ^24^ The PAVLOVA dataset includes data from both public and private hospitals, setting it apart from datasets that do not include private hospital admissions. Further, the data we analysed were recent compared with those of similar studies. The cohort size allowed us to stratify excess mortality by cause, including by body systems. Most years of life lost were among people under 55 years of age, and a median of 31.8 years of life were lost per death. Suicide deaths were responsible for 33.8% of years of life lost; accidental poisoning, accidental injury, and natural causes were the other major contributors. Our findings confirm that excess mortality after hospitalisation with intentional self‐poisoning remains a problem in New South Wales.

The frequency of intentional self‐poisoning by young women and girls has increased in Australia since 2011,^9^, ^14^ causing concern that this rise could be followed by an increase in the subsequent suicide death rate. In our study, the numbers of suicide and accidental poisoning deaths and the median number of associated years of life lost were greater for male than female former patients. However, as these two causes of death are generally less frequent among women than men (ie, the expected population rates are lower), the SMRs for suicide and accidental poisoning were higher for female than male former patients. If the intentional self‐poisoning rate for girls and young women continues to rise, the suicide and accidental poisoning death rates may also increase. This possibility should be examined in follow‐up studies, as much of the recent increase in the intentional self‐poisoning rate was reported in 2021,^14^ after our study period.

After adjusting for age and sex, people admitted to hospital after self‐poisoning were 25 times as likely to die from suicide as other people in the general population. Similar findings in other studies^1^, ^4^, ^6^, ^7^, ^24^ have been accompanied by different SMR estimates (suicide SMR range, 18.7 to 77; overall SMR range, 3.6 to 12.5), probably because of differences in study populations, follow‐up periods, and definitions of index events. Despite the greater risk of suicide death, we found that most people admitted to hospital after self‐poisoning did not die from suicide during follow‐up. Predicting who is at risk of death by suicide after self‐harm could facilitate targeted suicide prevention. Unfortunately, while repeated self‐harm, suicidal intent, and poor physical health are predictors of suicide risk, they are too common to be of practical use, as is the greater risk for men than women.^25^ Suicide prevention could instead focus on means restriction, as it reduces the incidence of suicide and does not require targeting of people with particular risk factors.^26^

The increase in mortality risk was greatest during the year after the index poisoning hospitalisation event, consistent with the findings of other studies.^4^, ^5^ This period is consequently an important time for interventions. A randomised controlled trial found that sending postcards to people after hospitalisation with intentional self‐poisoning reduced the number of repeat and psychiatric illness‐related admissions.^27^ Our findings suggest that the first two months are particularly important for targeted interventions.

Deaths from natural causes following non‐fatal intentional self‐harm have been less investigated than deaths from external causes. The increased risk of natural cause death is probably multifactorial, but primarily related to risk factors shared by intentional self‐poisoning and physical disease. People who intentionally harm themselves are more likely to have physical health risk factors, including smoking,^28^ heavy episodic alcohol use,^29^ and obesity.^30^ Certain psychotropic agents, including atypical antipsychotics, are often prescribed for people who intentionally harm themselves, and they can have adverse metabolic effects.^31^ In addition, social factors, including low socio‐economic status, are associated with both intentional self‐harm and physical disease, and people with severe mental illness are less likely to receive standard levels of care for physical diseases.^32^ While we do not believe that the link between intentional self‐poisoning and greater risk of natural death during follow‐up is causal, our findings have clinical implications, particularly the need to consider the physical needs of people who intentionally poison themselves.

The risk of death from accidental poisoning after hospitalisation with deliberate self‐poisoning was substantially higher than for the general population. However, deaths coded as accidental poisonings may have been the result of deliberate self‐poisoning. Miscoding could reflect uncertainty about the circumstances, the high level of proof required for a suicide determination, or the stigmatisation of suicide.^33^, ^34^ A United Kingdom study found that poisoning suicides were increasingly miscoded as accidental poisonings during 1990–2005.^35^ In addition, accidental poisoning codes capture deaths following recreational substance use, including heroin overdoses. While these events are often considered accidents, some people attempt suicide using recreational substances.^34^, ^36^ The higher risk of accidental poisoning is likely to reflect a combination of code shifting, events for which self‐harm intent could not be ascertained, and genuine accidental poisonings. Combined with the high proportion of people in our study with recorded ICD‐10‐AM codes for mental and behavioural disorders related to psychoactive substance use (44.5%), this finding underscores the need for brief interventions and referrals for people with drug and alcohol problems hospitalised because of self‐harm. Mental health assessments are part of standard care following self‐harm; screening for substance use problems could similarly be incorporated into routine care.

Only two similar studies have reported the mortality burden as years of life lost. Our estimate of 31.8 years of life lost per death was similar to those reported by a United Kingdom study (31.4 YLL)^1^ and a Finnish study (33.6 YLL).^6^ In our study, 33.8% of years of life lost were attributable to suicide deaths, mostly by hanging, and 21.8% to accidental poisonings. The most frequent natural causes by lost life years were ischaemic heart disease, chronic obstructive pulmonary disease, and alcoholic liver disease.

### Limitations

We defined the first event in the dataset as the index self‐poisoning hospitalisation, but it is possible that some people had been hospitalised for this reason before 2011. Further, we included only self‐poisonings that led to hospital admissions. Not all poisonings lead to admissions; some people are treated in emergency departments, and others do not present to hospital at all. As the poisoning cases we included were probably more severe than such cases, our findings may not be generalisable to people with medically less severe poisonings. Our comorbidity data were based on APDC diagnosis codes, and probably did not capture the full extent of multimorbidity in our cohort. Finally, code shifting means we may have underestimated the number of suicide deaths and overestimated that of deaths from accidental injuries. Code shifting in the APDC data is also possible, and some cases of intentional self‐poisoning may have been missed. The duration of follow‐up affects all estimates, as SMRs declined over time after self‐poisoning; longer follow‐up would lead to lower SMR values but higher YLL estimates.

### Conclusion

People who intentionally poison themselves require special care, as intentional self‐poisoning is usually associated with multiple life problems. Aftercare should not be limited to assessing suicide risk, but should also consider physical health needs and substance use problems. Continued monitoring of excess mortality after intentional self‐poisoning is warranted in order to assess birth cohort effects in women born since 2000, who are increasingly engaging in self‐harm.

## Competing interests

Rose Cairns has received an untied educational grant from Reckitt to fund a PhD stipend for paracetamol overdose research, unrelated to the investigation described in this article. Rose Cairns has also received honoraria from Reckitt and the Pharmacy Guild of Australia for presentations on over‐the‐counter medicines poisoning, unrelated to this article. The other authors declare no financial relationships with any organisations that might have an interest in the reported study.

## Data sharing

The PAVLOVA dataset cannot be shared for privacy reasons. Programming code is available on request.

## Author contributions

Conceptualisation: Rose Cairns. Methodology: Firouzeh Noghrehchi, Nicholas A Buckley. Formal analysis: Firouzeh Noghrehchi. Resources: Nicholas A Buckley, Rose Cairns. Writing (original draft): Rose Cairns. Writing (review and editing): Nicholas A Buckley, Firouzeh Noghrehchi. Visualisation: Firouzeh Noghrehchi. Project administration: Rose Cairns. Funding acquisition: Rose Cairns, Nicholas A Buckley.

## Supporting information


Supplementary methods and results
