# Supplementary material for: The risk of death after hospitalisation following intentional self‐poisoning: a retrospective observational study (PAVLOVA‐2)
Source: Med J Aust. 2025 Oct 8;223(11):634–42. doi: 10.5694/mja2.70068 (PMC12683317; doi:10.5694/mja2.70068)
Supplement: Supplementary file 1 — Supplementary methods and results [file MJA2-223-634-s001.pdf]

The Medical Journal of Australia

Supporting Information

Supplementary methods and results

This appendix was part of the submitted manuscript and has been peer reviewed. It is posted as supplied by the authors.

Appendix to: Noghrehchi F, Buckley NA, Cairns R. The risk of death after hospitalisation following intentional self-poisoning: a retrospective observational study (PAVLOVA-2). *Med J Aust* 2025; doi: 10.5694/mja2.70068.

Supplementary methods

Table 1. International Classification of Diseases, tenth revision, Australian modification (ICD-10-AM) codes for the classification of deaths

Natural causes: A00-R99
External causes: V01–Y89

Suicide: X60-X84
- By poisoning: X60-X69
- Violent methods: X70-X83
- Unspecified methods: X84

Accidental poisoning (X40-X49)
Assault (X85-Y09)
Accidental drowning (W65-W74)
Land transport accidents (V01-V89)

Table 2. Suicide means (ICD-10-AM code group) for the 644 suicide deaths following index self-poisoning hospital admissions

Suicide means	Male	Female	Total
**All means**	373 [57.9%]	271 [42.1%]	644
Intentional self-poisoning by and exposure to nonopioid analgesics, antipyretics and antirheumatics (X60)	<5	<5	<5
Intentional self-poisoning by and exposure to antiepileptic, sedative-hypnotic, antiparkinsonism and psychotropic drugs, not elsewhere classified (X61)	37 (9.9%)	28 (10.3%)	65 (10.1%)
Intentional self-poisoning by and exposure to narcotics and psychodysleptics [hallucinogens], not elsewhere classified (X62)	14 (3.8%)	8 (3.0%)	22 (3.4%)
Intentional self-poisoning by and exposure to other drugs acting on the autonomic nervous system (X63)	<5	<5	<5
Intentional self-poisoning by and exposure to other and unspecified drugs, medicaments and biological substances (X64)	40 (10.7%)	61 (22.5%)	101 (15.7%)
Intentional self-poisoning by and exposure to alcohol (X65)	<5	<5	<5
Intentional self-poisoning by and exposure to organic solvents and halogenated hydrocarbons and their vapours (X66)	<5	<5	<5
Intentional self-poisoning by and exposure to other gases and vapours (X67)	21 (5.6%)	5 (1.8%)	26 (4.0%)
Intentional self-poisoning by and exposure to pesticides (X68)	<5	<5	<5
Intentional self-poisoning by and exposure to other and unspecified chemicals and noxious substances (X69)	<5	<5	<5
Intentional self-harm by hanging, strangulation and suffocation (X70)	179 (48.0%)	107 (39.5%)	286 (44.4%)
Intentional self-harm by drowning and submersion (X71)	9 (2.4%)	7 (2.6%)	16 (2.5%)
Intentional self-harm by handgun discharge (X72)	<5	<5	<5
Intentional self-harm by rifle, shotgun and larger firearm discharge (X73)	<5	<5	<5
Intentional self-harm by other and unspecified firearm discharge (X74)	<5	<5	<5
Intentional self-harm by explosive material (X75)	<5	<5	<5
Intentional self-harm by smoke, fire and flames (X76)	<5	<5	6 (0.9%)
Intentional self-harm by steam, hot vapours and hot objects (X77)	<5	<5	<5
Intentional self-harm by sharp object (X78)	9 (2.4%)	10 (3.7%)	19 (3.0%)
Intentional self-harm by blunt object (X79)	<5	<5	<5
Intentional self-harm by jumping from a high place (X80)	18 (4.8%)	16 (5.9%)	34 (5.3%)
Intentional self-harm by jumping or lying before moving object (X81)	17 (4.6%)	13 (4.8%)	30 (4.7%)
Intentional self-harm by crashing of motor vehicle (X82)	10 (2.7%)	<5	11 (1.7%)
Intentional self-harm by other specified means (X83)	<5	<5	<5
Intentional self-harm by unspecified means (X84)	<5	6 (2.2%)	8 (1.2%)

ICD-10-AM = International Classification of Diseases, tenth revision, Australian modification.

Table 3. Suicide means (ICD-10-AM code group) for the 644 suicide deaths following index self-poisoning hospital admissions*

	Self-poisoning (X60-X69)		Violent and unspecified methods (X70-X84)	
**Number of deaths**	229		415	
**Age (years)**	**Male**	**Female**	**Male**	**Female**
<15	<5	<5	<5	<5
15-24	13	11	48	26
25-34	21	16	48	37
35-44	30	25	55	32
45-54	28	27	48	38
55-64	16	17	25	9
65-74	7	7	16	10
75-84	<5	<5	7	7
85+	<5	<5	5	<5

ICD-10-AM = International Classification of Diseases, tenth revision, Australian modification.

* This quantifies the degree of method switching; i.e. switching from a non-fatal index event (all were self-poisonings) to fatal intentional self-harm using other methods.

Figure 1. Mortality during follow-up after index hospital admission following non-fatal intentional self-poisoning, PAVLOVA cohort, New South Wales, 1 January 2011 – 30 September 2018: standardised mortality ratios (SMR), by cause of death and time since index admission (months)*

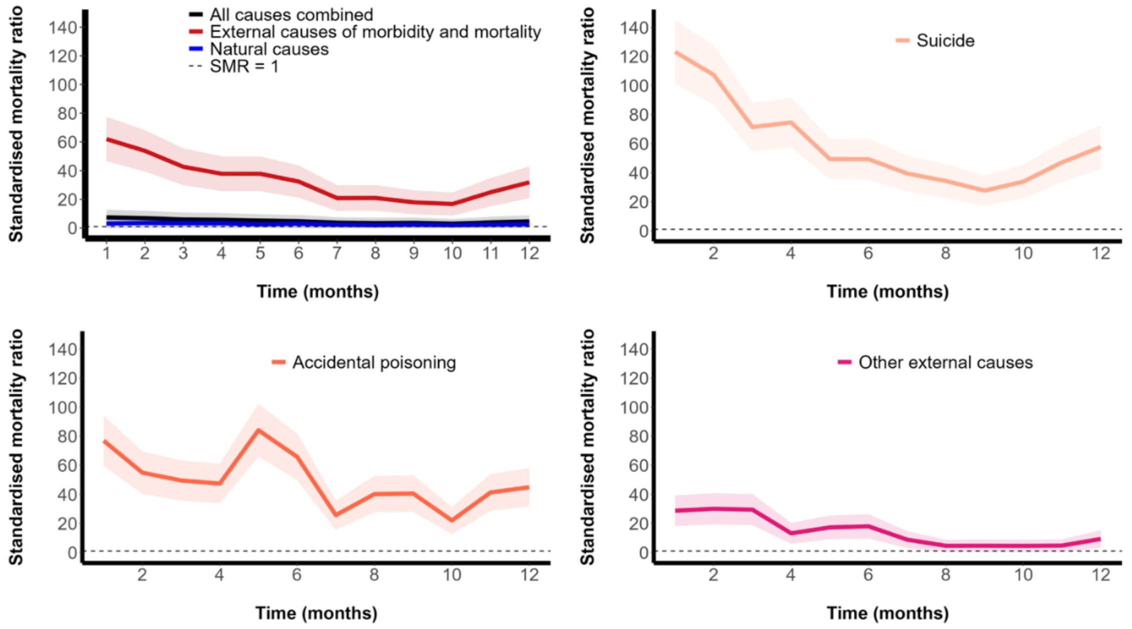

PAVLOVA = Poisoning And enVenomation Linkage to examine Outcomes and clinical Variation in Australia.
* Shaded areas: 95% confidence intervals; dotted lines mark SMR = 1 (comparative population level).

Figure 2. Mortality during follow-up after index hospital admission following non-fatal intentional self-poisoning, PAVLOVA cohort, New South Wales, 1 January 2011 – 30 September 2018: leading causes of years of life lost (YLL), by ICD-10-AM code groups and sex

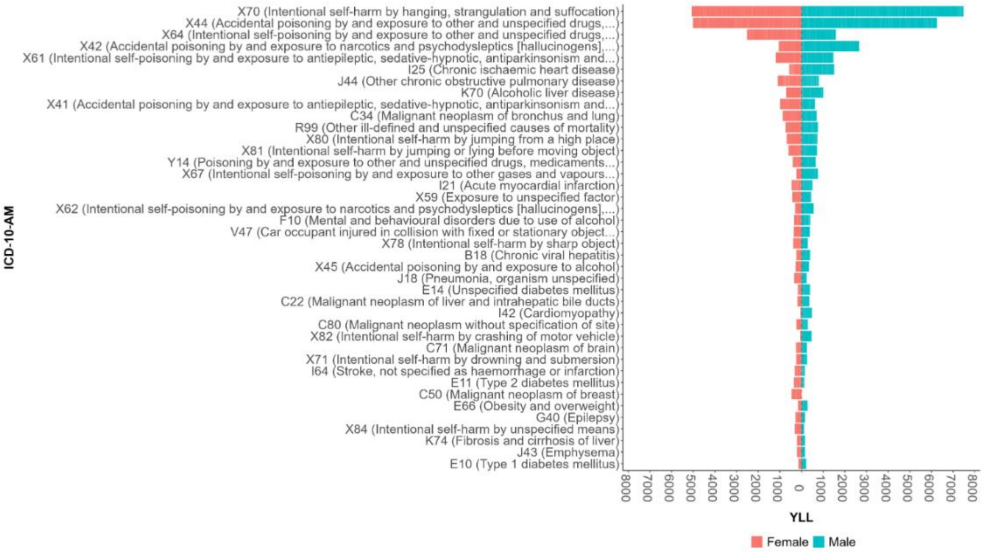

ICD-10-AM = International Classification of Diseases, tenth revision, Australian modification; PAVLOVA = Poisoning And enVenomation Linkage to examine Outcomes and clinical Variation in Australia.

Figure 3. Mortality during follow-up after index hospital admission following non-fatal intentional self-poisoning, PAVLOVA cohort, New South Wales, 1 January 2011 – 30 September 2018: total years of life lost (YLL), by ICD-10-AM code groups, age, and sex

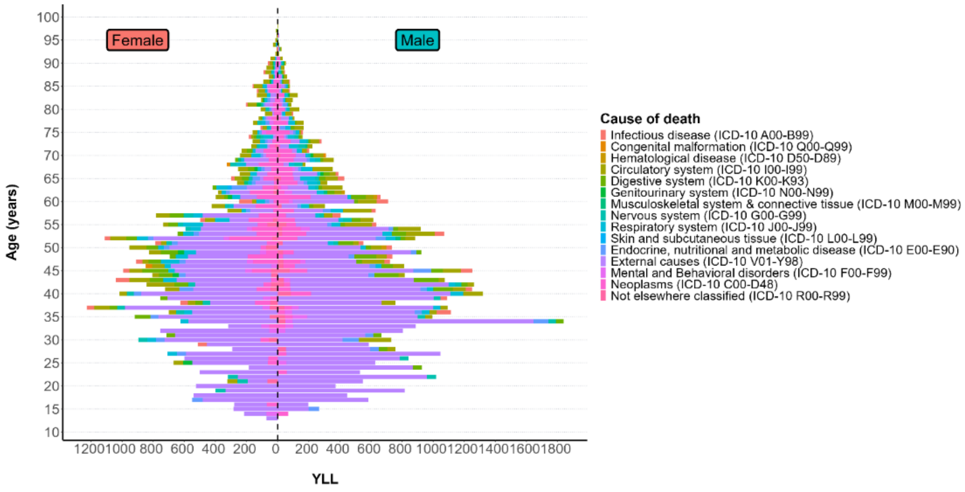

ICD-10-AM = International Classification of Diseases, tenth revision, Australian modification; PAVLOVA = Poisoning And enVenomation Linkage to examine Outcomes and clinical Variation in Australia.

Table 4. Mortality during follow-up after index hospital admission following non-fatal intentional self-poisoning, PAVLOVA cohort, New South Wales, 1 January 2011 – 30 September 2018: total years of life lost (YLL), by ICD-10-AM code groups and sex

Cause of death	Total YLL	Male YLL	Female YLL	Male-to-female ratio
Intentional self-harm by hanging, strangulation and suffocation (X70)	12658.8	7579.4	5079.4	1.5
Accidental poisoning by and exposure to other and unspecified drugs, medicaments and biological substances (X44)	11260.8	6252.9	5007.9	1.3
Intentional self-poisoning by and exposure to other and unspecified drugs, medicaments and biological substances (X64)	4108.2	1587.9	2520.3	0.6
Accidental poisoning by and exposure to narcotics and psychodysleptics [hallucinogens], not elsewhere classified (X42)	3713.7	2662.8	1050.9	2.5
Intentional self-poisoning by and exposure to antiepileptic, sedative-hypnotic, antiparkinsonism and psychotropic drugs, not elsewhere classified (X61)	2664.7	1472.0	1192.7	1.2
Chronic ischaemic heart disease (I25)	2079.2	1509.5	569.7	2.7
Other chronic obstructive pulmonary disease (J44)	1900.3	802.5	1097.8	0.7
Alcoholic liver disease (K70)	1712.9	1005.9	707.0	1.4
Accidental poisoning by and exposure to antiepileptic, sedative-hypnotic, antiparkinsonism and psychotropic drugs, not elsewhere classified (X41)	1613.7	620.9	992.8	0.6
Malignant neoplasm of bronchus and lung (C34)	1559.9	699.0	860.9	0.8
Other ill-defined and unspecified causes of mortality (R99)	1488.3	757.1	731.2	1.0
Intentional self-harm by jumping from a high place (X80)	1418.3	733.8	684.5	1.1
Intentional self-harm by jumping or lying before moving object (X81)	1316.5	717.3	599.2	1.2
Poisoning by and exposure to other and unspecified drugs, medicaments and biological substances, undetermined intent (Y14)	1070.8	655.8	415.0	1.6
Intentional self-poisoning by and exposure to other gases and vapors (X67)	983	751.3	231.7	3.2
Acute myocardial infarction (I21)	958	502.6	455.4	1.1
Exposure to unspecified factor (X59)	865.4	442.4	423.0	1.1
Intentional self-poisoning by and exposure to narcotics and psychodysleptics [hallucinogens], not elsewhere classified (X62)	863.7	560.7	303.0	1.9
Mental and behavioral disorders due to use of alcohol (F10)	744.5	401.3	343.2	1.2
Car occupant injured in collision with fixed or stationary object (V47)	733.8	380.2	353.6	1.1
Intentional self-harm by sharp object (X78)	672.3	285.6	386.7	0.7
Chronic viral hepatitis (B18)	638.5	398.7	239.8	1.7
Accidental poisoning by and exposure to alcohol (X45)	615	346.3	268.7	1.3
Pneumonia, organism unspecified (J18)	581.3	239.4	341.9	0.7
Unspecified diabetes mellitus (E14)	568.4	394.0	174.4	2.3
Malignant neoplasm of liver and intrahepatic bile ducts (C22)	545.5	359.4	186.1	2.0
Cardiomyopathy (I42)	530.3	479.2	51.1	9.4
Malignant neoplasm without specification of site (C80)	528.4	288.8	239.6	1.2
Intentional self-harm by crashing of motor vehicle (X82)	516.8	459.8	57.0	8.1
Malignant neoplasm of brain (C71)	507.4	250.1	257.3	1.0
Intentional self-harm by drowning and submersion (X71)	503.6	260.1	243.5	1.1
Stroke, not specified as hemorrhage or infarction (I64)	484.3	163.3	321.0	0.5
Type 2 diabetes mellitus (E11)	483.6	132.7	350.9	0.4

Cause of death	Total YLL	Male YLL	Female YLL	Male-to-female ratio
Malignant neoplasm of breast (C50)	459.8	0	459.8	N/A
Obesity and overweight (E66)	440.5	279.1	161.4	1.7
Epilepsy (G40)	440	159.0	281.0	0.6
Intentional self-harm by unspecified means (X84)	434.2	116.9	317.3	0.4
Fibrosis and cirrhosis of liver (K74)	374.7	165.6	209.1	0.8
Emphysema (J43)	371.2	161.3	209.9	0.8
Type 1 diabetes mellitus (E10)	353.9	212.8	141.1	1.5

ICD-10-AM = International Classification of Diseases, tenth revision, Australian modification; PAVLOVA = Poisoning And enVenomation Linkage to examine Outcomes and clinical Variation in Australia.

Figure 4. Mortality during follow-up after index hospital admission following non-fatal intentional self-poisoning, PAVLOVA cohort, New South Wales, 1 January 2011 – 30 September 2018: total years of life lost (YLL), by ICD-10-AM code groups and age

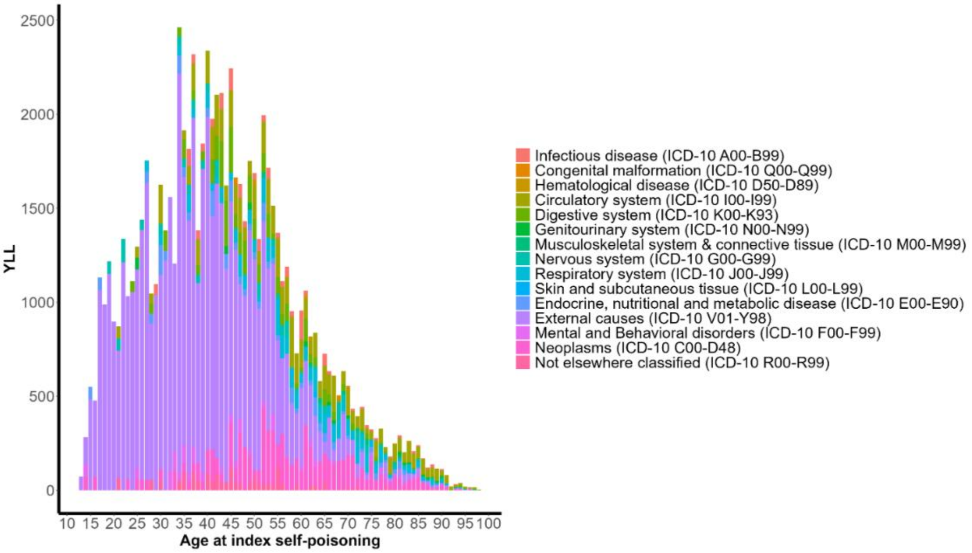

ICD-10-AM = International Classification of Diseases, tenth revision, Australian modification; PAVLOVA = Poisoning And enVenomation Linkage to examine Outcomes and clinical Variation in Australia.

Figure 5. Mortality during follow-up after index hospital admission following non-fatal intentional self-poisoning, PAVLOVA cohort, New South Wales, 1 January 2011 – 30 September 2018: total years of life lost (YLL), by age, sex, and broad cause of death category

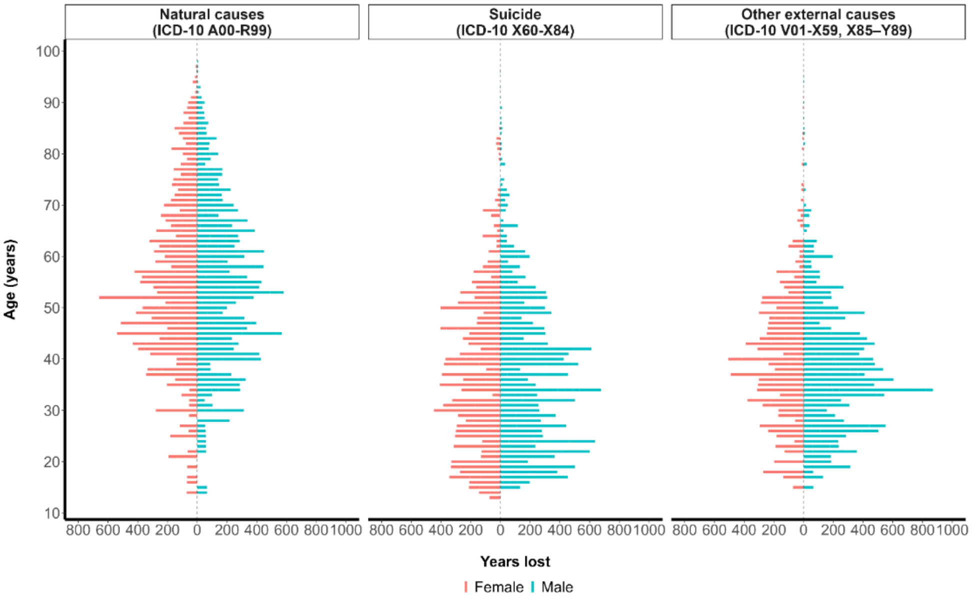

ICD-10-AM = International Classification of Diseases, tenth revision, Australian modification; PAVLOVA = Poisoning And enVenomation Linkage to examine Outcomes and clinical Variation in Australia.

The RECORD statement – checklist of items, extended from the STROBE statement, that should be reported in observational studies using routinely collected health data.

	Item No.	STROBE items	Location in manuscript where items are reported	RECORD items	Location in manuscript where items are reported
**Title and abstract**					
	1	(a) Indicate the study's design with a commonly used term in the title or the abstract (b) Provide in the abstract an informative and balanced summary of what was done and what was found	Title, abstract	RECORD 1.1: The type of data used should be specified in the title or abstract. When possible, the name of the databases used should be included.<br><br>RECORD 1.2: If applicable, the geographic region and timeframe within which the study took place should be reported in the title or abstract.<br><br>RECORD 1.3: If linkage between databases was conducted for the study, this should be clearly stated in the title or abstract.	Linked data (title)<br>Database names (abstract)<br><br>Abstract<br><br>Title, abstract
**Introduction**					
Background rationale	2	Explain the scientific background and rationale for the investigation being reported	Introduction		
Objectives	3	State specific objectives, including any prespecified hypotheses	Introduction, aims statement		
**Methods**					
Study Design	4	Present key elements of study design early in the paper	Top section of methods		
Setting	5	Describe the setting, locations, and relevant dates, including periods of recruitment, exposure, follow-up, and data collection	Methods: design and datasets		
Participants	6	*(a) Cohort study* - Give the eligibility criteria, and the sources and methods of selection of participants. Describe methods of follow-up<br>*Case-control study* - Give the eligibility criteria, and the sources and methods of case ascertainment and control selection. Give the rationale for the choice of cases and controls<br>*Cross-sectional study* - Give the eligibility criteria, and the sources and methods of selection of participants<br><br>*(b) Cohort study* - For matched studies, give matching criteria and number of exposed and unexposed<br>*Case-control study* - For matched studies, give matching criteria and the number of controls per case	Methods: participants<br>Methods: analysis	RECORD 6.1: The methods of study population selection (such as codes or algorithms used to identify subjects) should be listed in detail. If this is not possible, an explanation should be provided.<br><br>RECORD 6.2: Any validation studies of the codes or algorithms used to select the population should be referenced. If validation was conducted for this study and not published elsewhere, detailed methods and results should be provided.<br><br>RECORD 6.3: If the study involved linkage of databases, consider use of a flow diagram or other graphical display to demonstrate the data linkage process, including the number of individuals with linked data at each stage.	ICD-10 X60 – X69 (methods). Codes for specific causes of death listed in tables.<br><br>N/A<br><br>Not done- this study used a simple linkage of 2 datasets only
Variables	7	Clearly define all outcomes, exposures, predictors,	Methods: analysis	RECORD 7.1: A complete list of codes and algorithms used to classify	Methods: analysis

		potential confounders, and effect modifiers. Give diagnostic criteria, if applicable.		exposures, outcomes, confounders, and effect modifiers should be provided. If these cannot be reported, an explanation should be provided.	
Data sources/ measurement	8	For each variable of interest, give sources of data and details of methods of assessment (measurement). Describe comparability of assessment methods if there is more than one group	Methods: analysis		
Bias	9	Describe any efforts to address potential sources of bias	N/A		
Study size	10	Explain how the study size was arrived at	N/A		
Quantitative variables	11	Explain how quantitative variables were handled in the analyses. If applicable, describe which groupings were chosen, and why	Methods: analysis		
Statistical methods	12	(a) Describe all statistical methods, including those used to control for confounding<br>(b) Describe any methods used to examine subgroups and interactions<br>(c) Explain how missing data were addressed<br>(d) *Cohort study* - If applicable, explain how loss to follow-up was addressed<br>*Case-control study* - If applicable, explain how matching of cases and controls was addressed<br>*Cross-sectional study* - If applicable, describe analytical methods taking account of sampling strategy<br>(e) Describe any sensitivity analyses	Methods: analysis		
Data access and cleaning methods		..		RECORD 12.1: Authors should describe the extent to which the investigators had access to the database population used to create the study population.<br><br>RECORD 12.2: Authors should provide information on the data cleaning methods used in the study.	Published separately in data linkage cohort description (referenced in this paper)
Linkage		..		RECORD 12.3: State whether the study included person-level, institutional-level, or other data linkage across two or more databases. The methods of linkage and methods of linkage quality evaluation should be provided.	Methods, with full details in the referenced cohort profile.
**Results**					
Participants	13	(a) Report the numbers of individuals at each stage of the study (*e.g.*, numbers potentially eligible, examined for eligibility, confirmed eligible, included in the study, completing follow-up, and analysed)<br>(b) Give reasons for non-participation at each stage.	First section of results.	RECORD 13.1: Describe in detail the selection of the persons included in the study (*i.e.,* study population selection) including filtering based on data quality, data availability and linkage. The selection of included persons can be described in the text and/or by means of the study flow diagram.	Methods

		(c) Consider use of a flow diagram			
Descriptive data	14	(a) Give characteristics of study participants (*e.g.*, demographic, clinical, social) and information on exposures and potential confounders<br>(b) Indicate the number of participants with missing data for each variable of interest<br>(c) *Cohort study* - summarise follow-up time (*e.g.*, average and total amount)	a)/b) table 1<br><br>c) results text		
Outcome data	15	*Cohort study* - Report numbers of outcome events or summary measures over time<br>*Case-control study* - Report numbers in each exposure category, or summary measures of exposure<br>*Cross-sectional study* - Report numbers of outcome events or summary measures	Results table 3		
Main results	16	(a) Give unadjusted estimates and, if applicable, confounder-adjusted estimates and their precision (e.g., 95% confidence interval). Make clear which confounders were adjusted for and why they were included<br>(b) Report category boundaries when continuous variables were categorized<br>(c) If relevant, consider translating estimates of relative risk into absolute risk for a meaningful time period	Results, tables		
Other analyses	17	Report other analyses done—e.g., analyses of subgroups and interactions, and sensitivity analyses	Results		
**Discussion**					
Key results	18	Summarise key results with reference to study objectives	Discussion first paragraph		
Limitations	19	Discuss limitations of the study, taking into account sources of potential bias or imprecision. Discuss both direction and magnitude of any potential bias	Discussion limitations paragraph	RECORD 19.1: Discuss the implications of using data that were not created or collected to answer the specific research question(s). Include discussion of misclassification bias, unmeasured confounding, missing data, and changing eligibility over time, as they pertain to the study being reported.	Misclassification discussed Limitations, selection issues & coding limitations discussed.
Interpretation	20	Give a cautious overall interpretation of results considering objectives, limitations, multiplicity of analyses, results from similar studies, and other relevant evidence	Discussion: conclusion		
Generalisability	21	Discuss the generalisability (external validity) of the study results	Discussion para 2		
**Other Information**					
Funding	22	Give the source of funding and the role of the funders for the present study and, if applicable, for the original	Scholar One		

		study on which the present article is based			
Accessibility of protocol, raw data, and programming code		..	Scholar One	RECORD 22.1: Authors should provide information on how to access any supplemental information such as the study protocol, raw data, or programming code.	Scholar One

* Benchimol EI, Smeeth L, Guttmann A, et al; RECORD Working Committee. The REporting of studies Conducted using Observational Routinely-collected health Data (RECORD) statement. PLoS Med 2015;12: e1001885.
Checklist is protected under Creative Commons Attribution (CC BY) license.
